# RETRACTED: Hardan et al. Variability in Enteric Methane Emissions among Dairy Cows during Lactation. *Animals* 2023, *13*, 157

**DOI:** 10.3390/ani16081140

**Published:** 2026-04-09

**Authors:** Ali Hardan, Philip C. Garnsworthy, Matt J. Bell

**Affiliations:** 1 School of Biosciences, The University of Nottingham, Sutton Bonington Campus, Loughborough LE12 5RD, UK; phil.garnsworthy@nottingham.ac.uk; 2 Animal and Agriculture Department, Hartpury University, Gloucester GL19 3BE, UK; matt.bell@hartpury.ac.uk

The journal retracts the article “Variability in Enteric Methane Emissions among Dairy Cows during Lactation” [1], cited above.

Following publication, concerns were brought to the attention of the Editorial Office regarding overlap between this publication [1] and an earlier article [2] published by a similar authorship group.

Adhering to our complaints procedure, an investigation was conducted by the Editorial Office and Editorial Board which included an evaluation of the scientific content and findings of the paper. While the authors fully cooperated with the investigation through the clarification of the raised concerns, the Editorial Board confirmed a significant overlap between these publications [1,2], without the appropriate acknowledgement and citation. Given the scale of the overlap, the Editorial Office and the Editorial Board consider this article [1] to be redundant and have decided to retract this article as per MDPI’s retraction policy (https://www.mdpi.com/ethics#_bookmark30, accessed on 22 March 2026).

This retraction was approved by the Editor-in-Chief of the journal *Animals*.

The authors did not agree with this retraction.
